# Role of neutrophil / lymphocyte ratio, uric acid / albumin ratio and uric acid / creatinine ratio as predictors to severity of preeclampsia

**DOI:** 10.1186/s12884-023-06083-6

**Published:** 2023-10-30

**Authors:** Rayan Abdelraheem Mohamed, Ibrahim Abdelrhim Ali

**Affiliations:** Department of Physiology, Faculty of Medicine, The National Ribat University, Khartoum, Sudan

**Keywords:** Preeclampsia, Neutrophil/Lymphocyte ratio, Uric acid/Creatinine ratio, Uric acid/ albumin ratio

## Abstract

**Background:**

Pre-eclampsia (PE) is an intractable obstetric disorder with high mortality and morbidity, affecting 6–8% of pregnancies worldwide. As its etiology and pathogenesis remain unclear, there are no specific prevention or treatment options. This study aimed to determine the association between neutrophil to lymphocyte ratio (NLR), uric acid to albumin ratio (UAR) and uric acid to creatinine ratio (UA/Cr) and severity of pre-eclampsia.

**Methods:**

A cross-sectional hospital-based study was conducted among pre-eclamptic women in Kosti Maternity Hospital from September to December 2022. Forty-five pre-eclamptic women were enrolled in this study and were classified according to the WHO classification of PE into mild PE (23 PE patients) and severe PE (22 PE patients). Data were collected using a semi-structured questionnaire covering medical history and clinical assessment. A blood sample was taken from each participant for measurements of the complete blood count (CBC), liver functions test (LFT) with enzymes, renal functions test (RFT) with electrolytes, and uric acid by standard techniques.

**Results:**

NLR was found to be statistically significantly higher in mothers with severe PE (6.3–9.9) than in those with mild PE (2.2–1.5) (p-value 0.048). Alanine aminotransferase (ALT) was significantly higher in women with severe PE than in those with mild PE (p-value = 0.02). The total means of platelet-lymphocyte ratio (PLR), UA/Cr, and UAR were insignificantly higher in women with severe PE compared with those with mild PE (p-values 0.666, 0.427, and 0.525, respectively). The means of uric acid and serum creatinine showed insignificant statistical elevation in women with severe PE compared with mild PE (p-values of 0.27 and 0.44, respectively). Serum albumin was found to be insignificantly lower in mothers with severe PE (3.3 ± 0.6 g/dl) than in those with mild PE (3.6 ± 0.6 g/dl); p-value = 0.21.

**Conclusions:**

PE showed a significant statistical increase in WBC, neutrophils, alanine transaminase (ALT), and NLR in severe PE compared to mild PE and a significant statistical decrease in lymphocyte count in severe PE compared to mild PE. The measurement of NLR may be a useful laboratory marker for predicting the severity of PE.

## Background

Hypertension was the second most common direct obstetric cause of maternal death worldwide (14%) [1]. Preeclampsia contributes 10–15% of maternal deaths worldwide. It is a multifactorial and complex disorder with genetic, environmental, immunological, and nutritional factors all playing a role in its pathogenesis, although the exact cause remains largely controversial [2].

Pre-eclampsia (PE) is an intractable obstetric disorder affecting 6–8% of pregnancies worldwide. PE is characterized by hypertension (blood pressure equal to or greater than 140/90mmHg), proteinuria (equal to or greater than 0.3 g/d), edema, and other symptoms. It can begin as early as 20 weeks of gestation and last for 6 weeks after delivery. In addition, PE is associated with high mortality and morbidity [3].

Pre-eclampsia is clinically classified by the WHO as mild or severe [4]. In most settings, pre-eclampsia is classified as severe if one of the following conditions is present: severe hypertension, severe proteinuria, or significant maternal organ dysfunction [4]. Whereas the American College of Obstetricians and Gynecologists (ACOG) in 2020 classified PE into mild PE with BP ≥ 140\90 on 2 occasions at least 6 h apart and proteinuria ≥ 300 mg/24 h, but without evidence of organ damage in the patient, and severe PE with the presence of one or more of the following symptoms and signs: BP of ≥ 160\110 mmHg on 2 occasions at least 6 h apart, proteinuria of more than 5 g in a 24-hour collection or more than 3 + in 2 random urine samples collected at least 4 h apart, pulmonary edema or cyanosis, oliguria, headache, epigastric pain, and thrombocytopenia [5]. The National Institute for Health and Care Excellence (NICE) has classified PE into two categories according to blood pressure: hypertension with a blood pressure of 140\90 to 159\109 mmHg and severe hypertension with a blood pressure of 160\110 mmHg [6].

The underlying etiology of pre-eclampsia is not well understood. A widely accepted cause of PE is based on the theory that abnormal placentation leads to abnormal remodeling of the spiral arteries, placental ischemia, hypoxia, and oxidative stress, all of which result in significant maternal physiological dysfunction [7]. Maternal circulating leukocytes are known to be involved in the pathogenesis of pre-eclampsia.

Maternal circulating leukocytes are activated in pregnancy and further activated in PE. All major leukocyte classes are activated, including neutrophils, lymphocytes, and monocytes. Neutrophils infiltrate the systemic vascular tissue in women with PE, causing vascular inflammation [8].

The syndrome of hemolysis, elevated liver enzymes, and low platelet count (HELLP syndrome) is a serious complication of PE. Mortality in women with HELLP syndrome is 0–24%, and perinatal death is up to 37% [9].

Platelets (PLT) may be involved in promoting an inflammatory response in PE through their interaction with leukocytes and endothelium as key inflammatory players. Accordingly, the platelet-to-lymphocyte ratio (PLR) (normal range of PLR is 1–2) and the neutrophil-to-lymphocyte ratio (NLR) (normal range of NLR is 0.78–3.53) have recently been used as systemic inflammatory response markers and may be options for clinical evaluation of PE [10].

Pre-eclampsia is considered a state of hyperuricemia due to a reduction in glomerular filtration rate [11]. Uric acid excretion is also impaired in PE as a result of lactate competition in the proximal tubule, while its production is magnified by increased trophoblastic turnover. It has been suggested that uric acid has a role in disease progression because its elevation leads to the inhibition of nitric oxide production, resulting in inadequate trophoblastic invasion and impaired endothelial repair, so it has been proposed as a predictor of disease severity [12].

Hypoalbuminemia in PE has been suggested as a predictor of disease severity. Hypoalbuminemia in PE is the result of reduced hepatic blood flow secondary to hypovolemia caused by increased capillary filtration pressure [13].

The uric acid to albumin ratio (UAR) has recently emerged as a marker of inflammation and oxidative stress and has good prognostic value in predicting clinical outcomes in patients with ST-segment elevation myocardial infarction (STEMI), It is also associated with the extent of coronary artery disease (CAD) in patients with acute coronary syndrome (ACS) and has also been shown to be a strong predictor of short-term mortality in patients with acute renal failure, but no previous study has been conducted to predict the relationship between the severity of PE and UAR [14]. (UAR was calculated by dividing the serum uric acid level by the serum albumin level) [14].

No previous studies have been conducted to predict the relationship between serum UA/Cr (UA/Cr was calculated by dividing serum uric acid by serum creatinine) and the severity of PE. Previous studies have shown an association between UA/Cr and PE severity, the presence of chronic obstructive pulmonary disease (COPD), lung function, hypertension, metabolic syndrome, type 2 diabetes, and malignancy [15].

As its etiology and pathogenesis remain unclear, there are no specific prevention or treatment options [16]. Delivery of the fetus and placenta has been described as the only effective treatment for PE [4]. The present study aimed to determine the association between neutrophil-to-lymphocyte ratio (NLR), uric acid-to-albumin ratio (UAR), and uric acid-to-creatinine ratio (UA/Cr) and the severity of pre-eclampsia.

## Materials and methods

This is a cross-sectional study conducted at Kosti Maternity Hospital from September to December 2022.

### Study area

Kosti Maternity Hospital is located in Kosti, White Nile State, in the southern part of Sudan, and it’s about 197 miles away from the capital of Sudan (Khartoum State). It’s a governmental hospital that’s affiliated with the Ministry of Health and provides obstetric and gynecological care. The hospital consists of two sections: the first is the gynecological section, with two wards consisting of 13 beds, and the second is the obstetric section, which involves antenatal, labor, and birth care, and postnatal wards with a capacity of 50 beds. The hospital is covered by 5 obstetric consultants, 3 anesthesia consultants, 9 obstetric specialists, 25 registrars, 30 house officers, 13 midwives, 20 nurses, 10 pharmacists, 9 technicians of anesthesia, and non-medical administrative staff as well as ancillary staff.

### Study population

Forty-five pre-eclamptic women were enrolled in this study and were classified into mild PE (23 PE patients) and severe PE (22 PE patients) according to the WHO classification [4] and ACOG classification [5]. The patients were classified as severe if any of the following conditions were present:

- Severe hypertension (diastolic blood pressure (DBP) ≥ 110 mmHg or systolic blood pressure (SBP) ≥ 160 mmHg on two occasions, six hours apart).
- Severe proteinuria ≥ 5 g/24 h.
- Significant maternal organ dysfunction (thrombocytopenia, severe persistent right upper quadrant or epigastric pain excluding all other alternative diagnoses, new-onset headache unresponsive to all forms of treatment, pulmonary edema, or renal insufficiency, and HELLP syndrome “hypertension, proteinuria, and presence of hemolytic anemia, elevated liver enzymes, and low platelet count”).

#### Inclusion criteria

- Women with preeclampsia (blood pressure ≥ 140/90 mmHg on 2 occasions, at least 6 h apart, and proteinuria of ≥ 300 mg/24 h in the second half of the pregnancy).

#### The exclusion criteria

- Pre-eclamptic women who refused to participate.
- Those with pre-existing liver disease, diabetes mellitus, renal failure, autoimmune disease, tuberculosis, heart disease, bone marrow disorders, and myeloproliferative disorders.

### Sampling method

Total coverage sampling was applied for those who agreed to participate in this study. The sample size was calculated using the equation n = Z(2) × P(P-1)/d(2) (n = sample size, Z = 1.96 for confidence level (confidence interval) up to 95%, P = estimated prevalence from previous studies, d = precision (margin of error) 5%, P = population) [17].

### Data collection and data collection tools

Written informed consent was signed by all participants. The data were collected using a semi-structured questionnaire. The questionnaire consists of demographic data (age, parity, and gestational age), clinical presentation (headache, epigastric pain, visual disturbance, jaundice, and lower limb edema), history of pre-eclampsia or medical condition, and family history of PE. Then five millimeters of venous blood were collected by clean venipuncture in a commercially prepared concentration of ethylene diamine tetraacetic acid (EDTA) anticoagulant by an automated method. The sample was run in a Sysmex KX-21 N hematology analyzer for detection of complete blood count (CBC), also in a lithium heparin anticoagulant tube, then a Mindray BS-200 clinical chemistry analyzer for detection of Liver functions test (LFT) with enzymes, Renal functions test (RFT) with electrolytes, and uric acid. Midstream urine was collected in a sterile urine container and color was observed using urine strips to detect proteinuria. All steps were performed using standard techniques.

### Data processing and analysis

The data were managed using the Statistical Package for Social Sciences (SPSS) software version 25. The data were collected, coded, categorized, and recorded in an Excel sheet. The data were then analyzed by SPSS, and they were presented and described using tables. Results were presented as mean ± SD and independent samples t-test was used to compare groups. A P-value of 0.05 was considered statistically significant.

## Results

The study enrolled a total of 45 pregnant women diagnosed with pre-eclampsia who attended the Kosti Maternity Hospital during the study period. The diagnosis was made according to the WHO criteria for PE, which is classified as severe if one of the following conditions is present: severe hypertension, severe proteinuria, or significant maternal organ dysfunction.

### Demographic data

Age, parity, and gestational age showed an insignificant statistical correlation with the severity of preeclampsia (Table 1).

#### Clinical presentations & risk factors

Regarding clinical presentation, headache, and lower limb edema were the more common symptoms in preeclampsia, (Table 2). Regarding risk factors, the majority of patients had a positive family history of preeclampsia, (Table 3). The blood pressure showed a statistically significant association with the severity of preeclampsia, (Table 4).

**Table 1:** Demographic data in women with mild and severe PE

Parameter	Total (n = 45)	Mild preeclampsia (n = 23)	Severe preeclampsia (n = 22)	P value
Age (year)	25.60 ± 6.5	26.09 ± 7.0	25.09 ± 6.2	0.632
Parity	1.71 ± 2.45	1.35 ± 2.33	2.09 ± 2.56	0.142
Gestational age (week)	34.38 ± 2.47	35.00 ± 2.36	33.73 ± 2.47	0.084

**Table 2:** Clinical presentation of preeclampsia *(n = 45)*

Symptom		Mild	Severe
Headache	Yes	15 (33.3%)	21 (46.7%)
	No	8 (17.8%)	1 (2.2%)
Visual disturbance	Yes	0 (0%)	3 (6.7%)
	No	23 (51.1%)	19 (42.2%)
Epigastric pain	Yes	2 (4.4%)	8 (17.8%)
	No	21 (46.7%)	14 (31.1%)
Jaundice	Yes	0 (0%)	4 (8.9%)
	No	23 (51.1%)	18 (40%)
Lower limb swelling	Yes	13 (28.9%)	11 (24.4%)
	No	10 (22.3%)	11 (24.4%)

**Table 3:** Risk factors of preeclampsia (n = 45)

Variable	Yes	No
Multiple gestation	5 (11.1%)	40 (88.9%)
Chronic HTN	5 (11.1%)	40 (88.9%)
Previous preeclampsia	1 (2.2%)	44 (97.8%)
Family history of preeclampsia	15 (33.3%)	30 (66.7%)

#### Hematological values

The variation of white blood cell (WBC), neutrophil count, and NLR count in pre-eclamptic women showed statistically significant differences, which are higher in women with severe preeclampsia. While the total mean lymphocyte count was statistically significant higher in women with mild PE than in women with severe PE, (Table 4).

Platelet lymphocyte ratio (PLR) was higher in women with severe PE compared with mild PE, but it had a statistically insignificant correlation with the severity of preeclampsia (Table 4).

MCHCs were higher in women with severe PE than in women with mild PE, which had a significant statistical correlation with the severity of preeclampsia (p-value = 0.026) (Table 5).

**Table 4:** Correlation of WBCs, neutrophils, lymphocyte, NLR, PLT and BP with mild and severe PE

Parameter	Total (n = 45)	Mild preeclampsia (n = 23)	Severe preeclampsia (n = 22)	P value
WBCs / (10^9/L)	9.92 ± 6.72	7.99 ± 2.79	11.94 ± 8.83	0.048*
Neutrophils (%)	61.02 ± 15.16	56.1 ± 12.26	66.2 ± 16.42	0.024*
Lymphocyte (%)	27.09 ± 11.34	30.43 ± 8.53	23.59 ± 12.97	0.042*
NLR	4.24 ± 7.3	2.2 ± 1.54	6.38 ± 9.99	0.048*
PLR	141.75 ± 254.59	111.39 ± 56.419	173.5 ± 361.11	0.666
Systolic BP / mmHg	156.22 ± 17.1	149.13 ± 11.25	163.64 ± 19.16	0.003*
Diastolic BP / mmHg	106.56 ± 14.05	96.30 ± 4.819	117.27 ± 12.41	0.000*

*p value of 0.05 which considered statistically significant

**Table 5:** Correlation between red cells and platelet parameter with mild and severe PE

Parameter	Total (n = 45)	Mild preeclampsia (n = 23)	Severe preeclampsia (n = 22)	P value
RBCs (10^12/L)	4.23 ± 0.83	4.07 ± 0.77	4.39 ± 0.88	0.192
Hb (g/dl)	11.46 ± 1.91	11.59 ± 2.22	11.32 ± 1.57	0.643
PCV(%)	34.68 ± 5.83	34.66 ± 6.15	34.71 ± 5.63	0.976
MCHC(g\dl)	33.59 ± 2.78	32.70 ± 1.88	34.53 ± 3.28	0.026*
PLT(10^9/L)	211.24 ± 86.93	228.87 ± 90.66	192.82 ± 80.78	0.167
PDW (fl.)	13.30 ± 2.34	13.44 ± 2.21	13.16 ± 2.5173	0.702
MPV (fl.)	9.09 ± 1.68	8.93 ± 1.61	9.26 ± 1.76	0.511

*p value of 0.05 which considered statistically significant

#### Biochemical values among PE patients

ALT was significantly higher in women with severe PE than in those with mild PE (p-value = 0.02). The mean of uric acid, UA/Cr, and UAR showed insignificant statistical correlations (p-values 0.427 and 0.525, respectively) (Table 6).

**Table 6:** Illustrate that renal function with electrolyte, uric acid, and liver function with enzyme

Parameter	Total (n = 25)	Mild preeclampsia (n = 23)	Severe preeclampsia (n = 22)	P value (T-Test)
Serum K (mmol/l)	3.96 ± 0.49	4.00 ± 0.54	3.91 ± 0.43	0.52
Serum Na (mmol/l)	140.84 ± 4.73	141.87 ± 4.26	139.77 ± 5.06	0.14
Urea (mg\dl)	41.64 ± 20.16	41.65 ± 11.85	41.64± 26.55	0.99
Creatinine (mg\dl)	1.1407 ± 20.16	1.02 ± 0.65	1.26 ± 1.30	0.44
Uric Acid (mg\dl)	6.87 ± 1.12	7.05 ± 0.89	6.68 ± 1.31	0.27
T-protein (g\dl)	6.67 ± 1.05	6.75 ± 1.17	6.59 ± 0.92	0.63
Albumin (g\dl)	3.48 ± 0.651	3.60 ± 0.65	3.36 ± 0.65	0.21
T.Bilirubin (mg\dl)	0.93 ± 0.738	0.96 ± 0.88	0.91 ± 0.59	0.84
ALP (U/L)	113.76 ± 45.93	109.39 ± 42.81	118.32 ± 49.57	0.52
AST (U/L)	44.31 ± 26.95	38.04 ± 10.95	50.86 ± 36.17	0.11
ALT (U/L)	34.9 ± 24.51	26.65 ± 9.51	43.52 ± 31.8	0.02*
UA/Cr	8.43 ± 4.61	8.87 ± 4.58	7.96 ± 4.7	0.427
UAR	2.1 ± 0.8	2.1 ± 0.9	2 ± 0.6	0.525

*p value of 0.05 which considered statistically significant

## Discussion

Hypertensive disorders during pregnancy are important health issues that need to be addressed, especially in developing countries where the incidence and rates of adverse outcomes are higher.

This study aimed to determine the association between neutrophil to lymphocyte ratio (NLR), uric acid to albumin ratio (UAR), and uric acid to creatinine ratio (UA/Cr) with the severity of pre-eclampsia among all pre-eclamptic women admitted to Kosti Maternity Hospital from September to December 2022.

In this study, 23 cases had mild pre-eclampsia and 22 cases had severe pre-eclampsia, according to the WHO classification of pre-eclampsia [4].

Age is a possible risk factor for PE. This study showed that the mean age group of PE was 25.6 ± 6.5 SD years, which is not consistent with previous studies done in Ethiopia, which illustrate that women over 35 have a 4–5 fold higher risk of PE compared to women aged 25–29 years [18].

This study showed that WBC and neutrophils were higher in women with severe PE than women with mild PE, while the lymphocyte count was higher in women with mild PE than with severe PE. These values were significant and consistent with the study done by Chomaw Sitotaw et al. [10], which showed that absolute lymphocyte count decreased in patients with pre-eclampsia compared to uncomplicated pregnancies. This finding may be explained by PE exaggerating the intravascular inflammatory response; platelet activation, leukocyte activation, and endothelial activation are exaggerated in preeclampsia compared to normal pregnancy, and their interaction is thought to result in the vascular damage in preeclampsia. The interaction of platelets with various cell types (endothelial, dendritic, T lymphocytes, neutrophils, and mononuclear phagocytes) may initiate and exaggerate inflammation in the arterial wall [10].

In this study, NLR was significantly increased in severe PE; this is in agreement with a few previous studies [8, 19]. This may be because neutrophils infiltrate systemic vascular tissue in women with pre-eclampsia, causing vascular inflammation [20]. Also, neutrophils were three times more numerous than lymphocytes per vessel [21]. The increase in NLR may also be explained by a previous study showing that patients with PE had significantly higher levels of C-reactive protein (CRP), interleukin (IL)-4, IL6, IL8, IL10, and tumor necrosis factor (TNF) than non-preeclamptic women and that higher levels of inflammatory biomarkers and white blood cells indicate more severe symptoms [22].

In this study, although the platelet/lymphocyte ratio (PLR) was higher in women with severe PE than in those with mild PE, it was not statistically significant, and this did not agree with the study by Priyanka Gogoi et al. [19]. Another study also reported a moderately negative correlation between maternal PLR and adverse neonatal outcomes, and they also found that NLR and PLR were significantly higher in neonates of mothers with PE [23].

This study showed that the mean of hemoglobin, erythrocytes, and PCV had a statistically insignificant correlation with the severity of pre-eclampsia, which agrees with the study obtained by Nahla Hwaitalla et al. [24], while MCHC was significantly higher in women with severe PE than mild PE, which also agrees with the study obtained by Nahla Hwaitalla et al. [24].

In this study, platelet count is higher in women with mild PE than severe PE; this indicates platelet count decreases with the severity of preeclampsia, but it is statistically insignificant compared with previous studies that illustrate that platelet count decreases in preeclampsia [10, 25]. Platelet distribution width (PDW) and mean platelet volume (MPV) were 13.3 ± 2.3 fl. SD and 9 ± 1. 6 fl. SD, respectively, which are normal values and do not correlate with previous studies [10, 19]. The platelet activation in PE is related to the alternation of the coagulation process between platelets and endothelial cells, thrombopoietin increases in patients with preeclampsia, so MPV and PDW are increased in preeclampsia more than in normal pregnant women. There is an active turnover of platelet production in the bone marrow, which results in continuous consumption of platelets in the peripheral blood, so platelet counts decrease below the normal range as preeclampsia progresses and PDW increases, so PDW may play a role as a predictive marker for the severity of preeclampsia before platelet counts are observed [26].

In terms of kidney function, creatinine and urea levels are not statistically significant with pre-eclampsia severity. This result is not consistent with the concept that the glomerular filtration rate (GFR) decreases in pre-eclampsia. This result does not agree with the study by Remah Mohaoud Abdelrahman et al., who showed that urea levels are increased in pre-eclampsia [27]. Increased urea levels in pre-eclamptic patients may be explained to some extent by the occurrence of microangiopathic hemolysis due to endothelial injury in pre-eclampsia, leading to increased urea synthesis in the liver, with the kidney unable to excrete urea from the blood; another possible explanation is that urea excretion is dependent on renal blood flow (RBF) and glomerular filtration, with less tubular reabsorption [27].

As a consequence of pre-eclampsia, hypovolemia leads to high angiotensin II and an angiotensin II hypersensitive state, and efferent arteriolar resistance and filtration are increased. Thus, angiotensin II enhances direct tubular reabsorption of water as well as urea, which may explain the elevated levels observed in pre-eclampsia [27]. A previous study showed that the incidence of AKI in women with severe pre-eclampsia was 42.86% [28].

In our results, creatinine was not statistically different between mild and severe pre-eclampsia. In another study not in agreement with our findings, Lindita Ibrahimi et al. showed that creatinine and the calcium-creatinine ratio contributed to the prediction of pre-eclampsia with an accuracy of 87.9% and could be used to predict the development of pre-eclampsia in asymptomatic patients. Women with a calcium-creatinine ratio of 0.105 have a higher risk of developing pre-eclampsia, and this ratio is inferior to protein and uric acid in predicting pre-eclampsia [29].

This study showed that electrolyte levels (sodium and potassium) were within the normal range and had an insignificant statistical correlation with the severity of PE, which is in agreement with a previous study [27] that showed normal plasma sodium and potassium levels in pre-eclamptic and normal pregnant women. In PE, the volume of circulating plasma is reduced, which may account for reduced Na + delivery to the distal nephron and reduced K + secretion [27]. These findings do not agree with a previous study, which showed that sodium and potassium levels were reduced in PE compared with normotensive pregnant women [30].

The mean serum uric acid was found to be lower in severe PE than in mild PE, but not statistically significant. In all PE patients, the mean serum uric acid was 6.87 ± 1.12 mg/dl, which is higher than the normal level. The increase in uric acid levels in pre-eclampsia has been reported previously [11, 12]. They suggested that serum uric acid levels are elevated in pre-eclampsia and can be used to predict disease severity and pregnancy complications [12]. Despite the formation of high levels of uric acid, increased free radical formation and oxidative stress in pre-eclampsia have been attributed to tissue ischemia [27]. Maternal serum uric acid level is an important parameter for predicting low birth weight, as shown by a 2019 study that showed that the maternal serum uric acid threshold for predicting low birth weight at delivery was 6.35 mg/dL [31]. A previous study showed that uric acid levels could be a potential management biomarker for immediate or delayed delivery in severe PE. Higher stillbirth rates were seen in cases of delayed delivery [32]. A study by Leticia G. Paula et al. showed that patients with eclampsia had higher serum uric acid levels and protein excretion, higher systolic and diastolic blood pressure, were more likely to have a caesarean section, and had worse perinatal outcomes [33]. A previous study showed that serum uric acid was significantly increased after 20 weeks’ gestation in women who developed PE before 34 weeks or in those who developed PE after 37 weeks in association with intrauterine growth restriction [34].

This study shows that the mean uric acid to creatinine ratio (UA/Cr) was 8.4 ± 4.6 SD with no significant statistical correlation (P value 0.427). This does not agree with a study by Federica in 2023, which showed that a higher UA/Cr ratio during pregnancy is associated with the development of PE and adverse pregnancy outcomes [35]. It also doesn’t agree with another study, which showed that the uric acid/creatinine ratio (UA/Cr) was higher and statistically significant in pre-eclamptic women [23]. Previous studies have shown the association of UA/Cr with the severity of pulmonary embolism, the presence of COPD, lung function, hypertension, metabolic syndrome, type 2 diabetes, and malignancy [15].

This study showed that the mean values of albumin and total protein were not statistically significantly correlated with the severity of pre-eclampsia, which is not consistent with the previous study by M. Gojnic et al. [13], in which they found that all patients with severe PE had values below 3.0 gm/dl, which may serve as an indicator of the severity of PE, and they propose that hypoalbuminemia in pre-eclampsia is the result of reduced hepatic blood flow secondary to hypovolemia caused by higher infiltration pressure in the capillaries.

There were no previous studies on the relationship between uric acid albumin ratio (UAR) and the severity of PE. In this study the mean UAR was 2.1 ± 0.8 SD, with no significant statistical correlation (P value of 0.52). Previous studies were performed in STEMI patients and in patients with acute renal failure [14].

In our study, bilirubin showed an insignificant statistical correlation compared to a previous study that found that bilirubin levels were the most important biochemical marker of maternal mortality and were statistically higher in cases of jaundice [36], The lowest quintile of bilirubin levels is associated with an increased risk of poor maternal and fetal outcomes because bilirubin is a known antioxidant and, as such, is associated with a reduced risk of cardiovascular and respiratory disease [37].

Regarding liver enzymes, this study shows that the mean values of aspartate aminotransferase (AST) and alkaline phosphatase (ALP) were not significantly elevated in severe PE compared to mild PE. However, a statistically significant elevation of alanine transferase (ALT) was found in severe PE compared to mild PE. A previous study found that serum ALT and AST levels were higher in pre-eclamptic pregnant women compared to normotensive pregnant women [38], and this is consistent with our findings, especially for ALT.

### Limitations of the study

Our study had several limitations. Firstly, the sample size was relatively small and therefore could not represent the current situation in the whole country. Second, it was an observational cross-sectional design, so patients were not followed up, and longitudinal data and outcomes were lacking. Finally, the cut-off value as a prognostic marker should be evaluated in larger studies. On the other hand, this research is a first step towards new laboratory criteria that may lead to better diagnosis and management of PE, and, to our knowledge, it’s the first study in Sudan and probably in Africa to determine the association between neutrophil/lymphocyte ratio (NLR), uric acid/albumin ratio (UAR), and uric acid/creatinine ratio (UA/Cr) and severity of pre-eclampsia.

## Conclusions

This study concluded that pre-eclampsia showed a significant increase in TWBCs, neutrophils, MCHC, ALT, and NLR in women with severe PE compared with mild PE and a significant decrease in lymphocyte count in women with severe PE compared with mild PE. The measurement of NLR may be a useful laboratory marker for predicting the severity of pre-eclampsia. While UAR and UC/Cr showed an insignificant statistical correlation, which requires further study.

## Data Availability

Data generated in this study are available from the corresponding author upon reasonable request with a completed Materials Transfer Agreement, excluding the materials including personally identifiable information.

## List of abbreviations

ALT: Alanine transaminase
ACOG: American College of Obstetricians and Gynecologists
CBC: Compete blood count
DBP: Diastolic blood pressure
HEELP: Syndrome Hemolysis elevated liver enzymes and low platelet count
MCHC: Mean corpuscular hemoglobin concentration
MPV: Mean platelet volume
NLR: Neutrophil to lymphocyte ratio
NICE: National Institute for Health and Care Excellence
PE: Preeclampsia
PLR: Platelet-to-Lymphocyte ratio
PLT: Platelets
PDW: Platelet distribution width
SBP: Systolic blood pressure
TWBCs: Total White blood cells WBCs
UAR: Uric acid to albumin ratio
UA/Cr: Uric acid to creatinine ratio
WBCs: White blood cells WBCs
